# Differential Activities of the Two Closely Related Withanolides, Withaferin A and Withanone: Bioinformatics and Experimental Evidences

**DOI:** 10.1371/journal.pone.0044419

**Published:** 2012-09-04

**Authors:** Kirti Vaishnavi, Nishant Saxena, Navjot Shah, Rumani Singh, Kavyashree Manjunath, M. Uthayakumar, Shankar P. Kanaujia, Sunil C. Kaul, Kanagaraj Sekar, Renu Wadhwa

**Affiliations:** 1 Supercomputer Education and Research Centre, Indian Institute of Science, Bangalore, India; 2 National Institute of Advanced Industrial Science and Technology (AIST), Central 4, Tsukuba, Japan; 3 Graduate School of Life and Environmental Sciences, University of Tsukuba, Ibaraki, Japan; Biological Research Centre of the Hungarian Academy of Sciences, Hungary

## Abstract

**Background and Purpose:**

Withanolides are naturally occurring chemical compounds. They are secondary metabolites produced via oxidation of steroids and structurally consist of a steroid-backbone bound to a lactone or its derivatives. They are known to protect plants against herbivores and have medicinal value including anti-inflammation, anti-cancer, adaptogenic and anti-oxidant effects. Withaferin A (Wi-A) and Withanone (Wi-N) are two structurally similar withanolides isolated from *Withania somnifera*, also known as Ashwagandha in Indian Ayurvedic medicine. Ashwagandha alcoholic leaf extract (i-Extract), rich in Wi-N, was shown to kill cancer cells selectively. Furthermore, the two closely related purified phytochemicals, Wi-A and Wi-N, showed differential activity in normal and cancer human cells *in vitro* and *in vivo*. We had earlier identified several genes involved in cytotoxicity of i-Extract in human cancer cells by loss-of-function assays using either siRNA or randomized ribozyme library.

**Methodology/Principal Findings:**

In the present study, we have employed bioinformatics tools on four genes, i.e., mortalin, p53, p21 and Nrf2, identified by loss-of-function screenings. We examined the docking efficacy of Wi-N and Wi-A to each of the four targets and found that the two closely related phytochemicals have differential binding properties to the selected cellular targets that can potentially instigate differential molecular effects. We validated these findings by undertaking parallel experiments on specific gene responses to either Wi-N or Wi-A in human normal and cancer cells. We demonstrate that Wi-A that binds strongly to the selected targets acts as a strong cytotoxic agent both for normal and cancer cells. Wi-N, on the other hand, has a weak binding to the targets; it showed milder cytotoxicity towards cancer cells and was safe for normal cells. The present molecular docking analyses and experimental evidence revealed important insights to the use of Wi-A and Wi-N for cancer treatment and development of new anti-cancer phytochemical cocktails.

## Introduction

Ashwagandha *(Withania somnifera: Solanaceae)* is a popular herb used in traditional home medicine and remedies that have been in practice in India and its neighboring countries for thousands of years. Although trusted for its wide health benefits, the active principles of Ashwagandha effects have not been understood to a large extent. Only recently, few studies on cell and animal models have demonstrated some mechanisms of its anti-inflammatory, anti-cancer, anti-diabetic, anti-stress, anti-oxidant, neuroprotective and immuno-modulatory potentials [1]–[4]. The major constituents of extracts, from various parts of Ashwagandha, are steroidal alkaloids and lactones, a class of chemicals known as withanolides (steroidal lactones with ergaostane skeleton) [5]. The withanolides have C28 steroidal nucleus with C9 side chain, having six-membered lactone ring. So far, 12 alkaloids, 35 withanolides and several sitoindosides have been isolated, and their structures have been elucidated [6], [7]. Various alkaloids include withanine, somniferine, somnine, somniferinine, withananine, psuedo-withanine, tropine, psuedotropine, 3-α-gloyloxytropane, choline, cuscohygrine, isopelletierine, anaferine and anahydrine. Two acyl steryl glucoside viz. sitoindoside VII and sitoindoside VIII, two glycowithanoloids viz. sitoindoside IX or sitoindoside X have been isolated from the roots of *Withania sominifera* [5]. Many toxicological studies have demonstrated that Ashwagandha, in its reasonable dose, is a non-toxic, safe and edible herb. Although withaferin A (Wi-A) is present in abundance in roots and leaves of Ashwagandha, its amount seems to vary with the geographical niche of the plants (Wadhwa et al., unpublished data). In earlier studies, it was shown to be a radio-sensitizer and a suppressor of mouse Ehrlich ascites carcinoma growth [8]–[10]. More recent studies have demonstrated that Wi-A induces apoptosis in human cancer cells [11]–[14]. It was shown to inhibit Notch-1 signaling and downregulate pro-survival pathways, such as Akt/NF-kappa B/Bcl-2 [15]. In human leukemia and melanoma cells, Wi-A was shown to induce apoptosis by activating the p38MAPK signaling cascade, induction of ROS generation, caspase cleavage and loss of Bcl-2 [3], [16]. It suppresses inflammation through inhibition of NO production and iNOS expression by blocking Akt and NF-kappa B activities [17]. Withanolide sulfoxide from Ashwagandha roots completely suppress TNF-induced NF-kappa B activation followed by inhibition of cyclooxygenase and tumor cell proliferation [18]. Other bioactive properties of Wi-A include inhibition of chymotrypsin like proteosomal activity [12], inhibition of protein kinase C [19], inhibition of Akt and Raf-1 pathways resulting in tumor suppression by induction of apoptosis and cell adhesion [4]. The effect of Wi-A on human normal cells has not been investigated in these studies.

Recently, we prepared alcoholic extract of Ashwagandha leaves (i-Extract) and undertook its chemical characterization and biological activity in normal and cancer human cell culture models. i-Extract caused selective killing of cancer cells [20]–[22]. By loss-of-function approach in which human siRNA and ribozyme libraries were used to identify gene targets, the selective cancer cell killing property of i-Extract and Wi-N was suggested to involve gene targets including p53, mortalin, p21^WAF1^, Nrf2, ING1, CDKN1A, CDKN2B, NEK2, CDK5, BIRC3, TFAP2A, TPX2 and DDB2 [20]–[22]. Recently, by computational analyses, binding mechanisms of withanolides on NF-κB signaling pathway [23], proteasome degradation pathway [24] and their role in altering chaperone/co-chaperone interactions [25] were also reported. In the present study, we undertook a computational approach to investigate whether Wi-A and Wi-N target the same proteins. Furthermore, these studies were validated by cell culture approach.

## Results and Discussion

### Response of Cancer and Normal Human Cells to Withaferin A and Withanone

**Figure 1:**
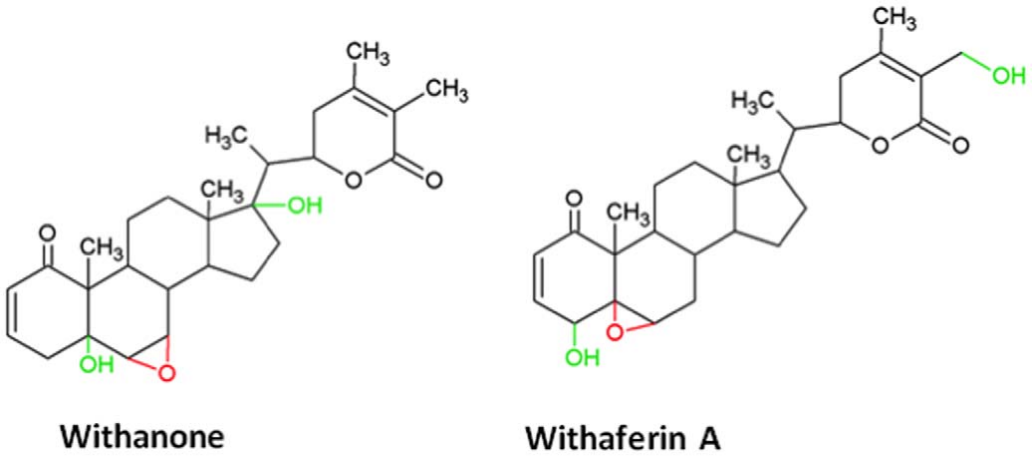
Chemical structures of withanone and withaferin A. Withanone is a C_6_, C_7_ epoxy compound (colored red) that has hydroxyl groups (colored green) on C_5_ and C_17_. On the other hand, withaferin A is a C_5_, C_6_ epoxy compound (colored red) with hydroxyl groups on C_4_ and C_27_ (colored green).

**Figure 2:**
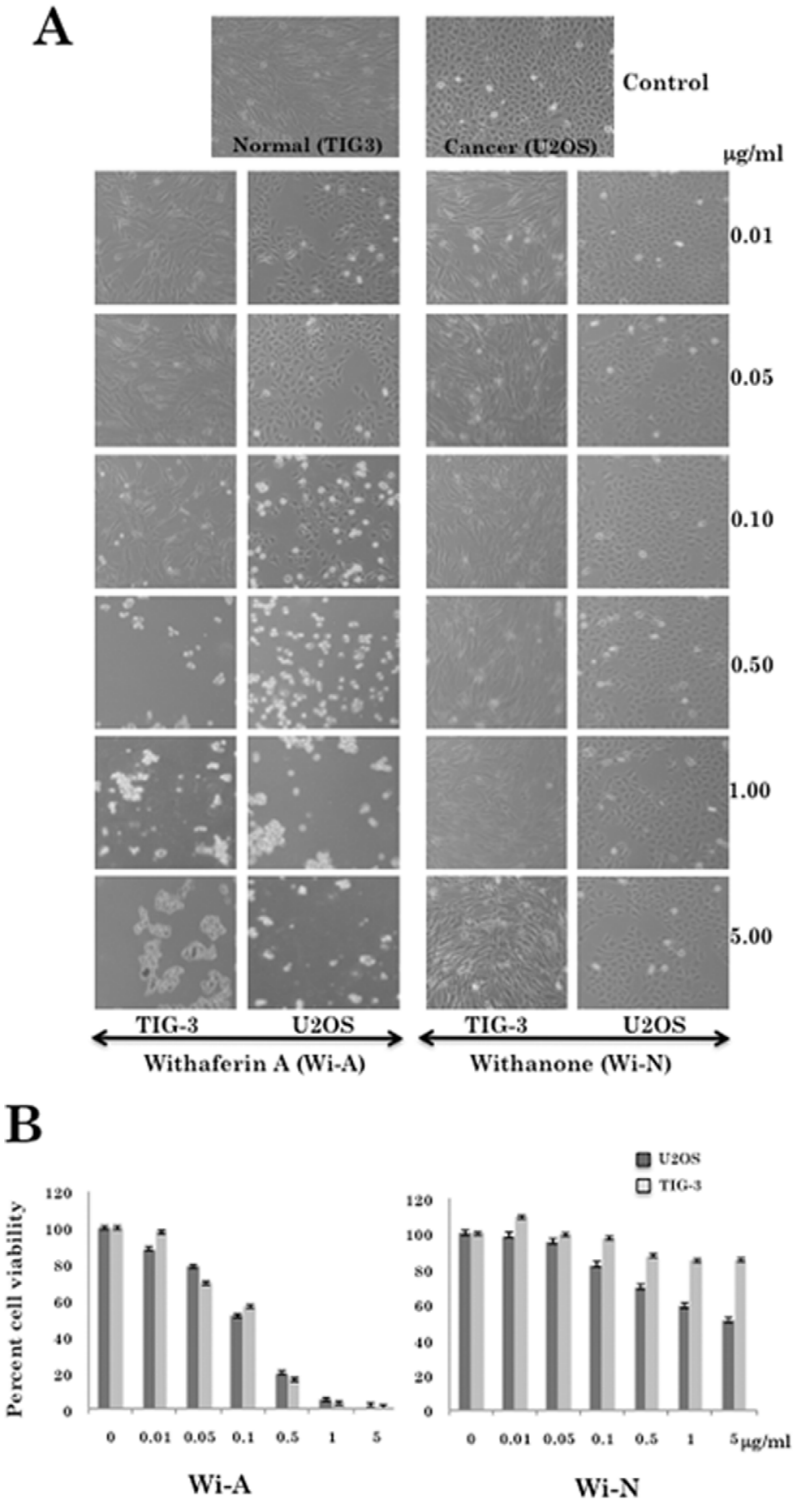
Effect of withanone and withaferin A on proliferation of human normal and cancer cells. (A) Morphology of the human normal (TIG) and cancer (U2OS) cells treated with different concentrations of withaferin A (Wi-A) and withanone (Wi-N). (B) MTT assay of control, Wi-A and Wi-N treated normal and cancer cells.

Withaferin A and withanone are the steroidal lactones with a withanolide-type A skeleton. Withanone is a C_6_, C_7_ epoxy compound that has hydroxyl groups on C_5_ and C_17_. On the other hand, withaferin A is a C_5_, C_6_ epoxy compound with hydroxyl groups on C_4_ and C_27_ (Figure 1). In addition, ADME (Absorption, Distribution, Metabolism and Excretion) analysis using QikProp software (Schrödinger suite) showed that both Wi-A and Wi-N are efficient (capable of exhibiting antagonistic and agonistic activities without having any side effects on the organism) small molecule compounds (Table 1). At this end, we treated human normal (TIG-3) and cancer (U2OS) cells either with withanone (Wi-N) or withaferin A (Wi-A) at concentrations ranging from 0.01–5.0 µg/ml. As shown in Figure 2A, we found that both normal and cancer cells were killed by Wi-A at doses 0.5 and above. Cells were rounded off and detached from culture dish and appeared like undergoing apoptosis. On the other hand, Wi-N treated cancer cells showed growth arrest; cells were enlarged and proliferated much slowly resulting in the decreased cell number as compared to the untreated control cells. Of note, Wi-N treated normal cells did not show growth arrest as evident by cell morphology and number. Cell viability as measured by MTT assay was consistent with these observations. As shown in Fig. 2B, Wi-A showed strong cytotoxicity to cancer as well as the normal cells. Wi-N, on the other hand, had milder effect; especially the normal cells remained unaffected. These data suggested that although Wi-A and Wi-N have closely related chemical structures, their biological activity and hence the drug-ability may be different. Accordingly, one needs to investigate the molecular mechanism(s) of their action (cellular targets and molecular response) for their use in therapeutics.

**Table 1:** ADME properties of Wi-A and Wi-N analyzed using QikProp.

Qikprop parameters	Withaferin A	Withanone
mol MW (130.0 – 725.0)	470.605	470.605
SASA (300.0 – 1000.0)	726.715	706.525
volume (500.0 – 2000.0)	1407.013	1383.253
DonorHB (0.0 – 6.0)	1	2
AccptHB (2.0 – 20.0)	9.4	8.5
QPlogS (−6.5 – 0.5)	−5.144	−5.402
QPlogBB (−3.0 – 1.2)	−1.41	−0.771
QPlogKp (−8.0 – 1.0)	−4.003	−3.034
PercentHumanOralAbsorption (>80% is high - 25% is poor)	86.573	100
RuleOfFive (max 4)	0	0

Mol MW: Molecular weight of the molecule.

SASA: Solvent accessible surface area.

donorHB: number of hydrogen bonds that would be donated.

accptHB: number of hydrogen bonds that would be accepted.

QPlogS: predicted aqueous solubility.

QPlogBB: predicted brain/blood partition coefficient.

QPlogKp: predicted skin permeability.

PercentHumanOralAbsorption: predicted human oral absorption on 0 to 100% scale.

RuleOfFive: number of violations of Lipinski’s rule of five.

In order to investigate the cellular targets of the alcoholic extract (i-Extract) of Ashwagandha leaves, rich in Wi-A and Wi-N, we previously adopted loss-of-function screenings using siRNA and ribozyme libraries in conjugation with the cell viability assays [20], [21]. These assays revealed the involvement of tumor suppressor protein p53 and oxidative stress pathways in i-Extract induced killing of cancer cells. In the present study, we investigated the docking characteristics of some of the gene targets (mortalin, p53, p21 and Nrf2) with Wi-A and Wi-N and validated the outcomes by cell-based molecular and imaging assays.

### Docking of Withaferin A and Withanone into Mortalin Structure and Experimental Examination of their Differential Effect in Human Cancer Cells

Mortalin is a stress chaperone of Hsp70 family of proteins that performs various functions related to proliferation, mitochondrial biogenesis, chaperoning and stress response [26], [27]. It is an essential protein that acts as a mitochondrial import motor and chaperone, mediates intracellular trafficking and is present at various subcellular sites including mitochondria, ER, plasma membrane and nucleus [27]–[29]. It has been shown to regulate tumor suppressor protein p53 by binding and sequestering it in the cytoplasm. Staining pattern of mortalin, perinuclear vs pancytoplasmic, has been shown to distinguish the cancer and normal cells, respectively. Cancer cells that were induced to senesce by introduction of chromosomes, chromosome fragments, peptides, chemicals and small molecules were shown to cause shift in mortalin staining pattern from perinuclear to pancytoplasmic type [27], [30] and release of p53 from mortalin-p53 complexes. Binding studies using deletion mutants have demonstrated that the N-terminal region of mortalin binds to the carboxy-terminus of the p53 protein [31], [32]. Most recently, it was shown that mortalin binds to the mutant p53 in cancer cells and inhibits its apoptotic functions. It, thus, acts as an anti-apoptotic factor contributing to the continued survival of cancer cells [33]. Since mortalin was identified as a target of i-Extract induced cancer cell death, we examined the interaction of mortalin with Wi-A and Wi-N by docking studies.

**Figure 3:**
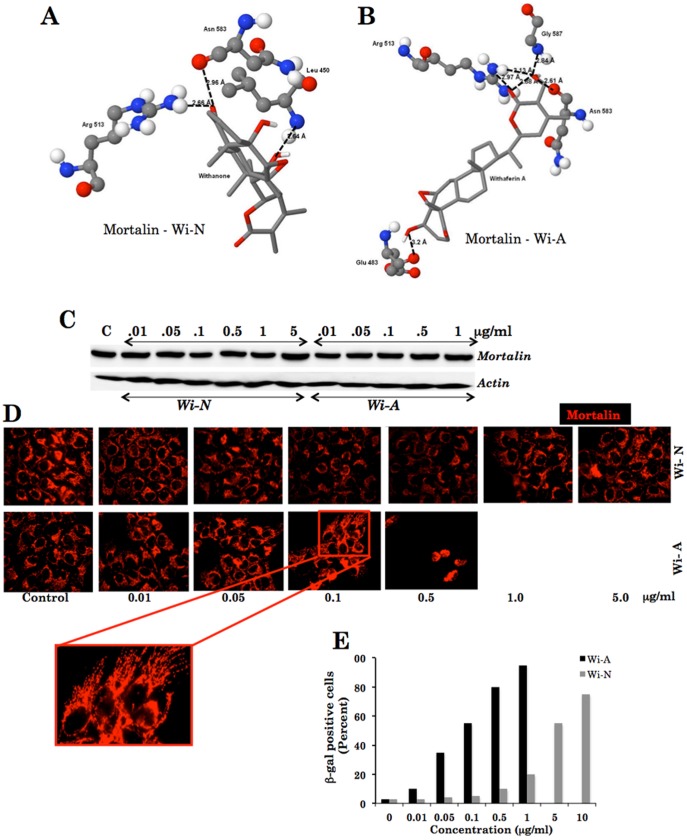
Mortalin, a target of withanone and withaferin A. Docking of withanone (Wi-N) (A) and withaferin A (Wi-A) (B) in the “latch region” of mortalin (PDB-id: 3N8E). (C) Quantitative analysis of mortalin in human cancer (U2OS) cells treated with various doses of Wi-N and Wi-A. (D) Imaging of mortalin in human cancer cells treated with various concentrations of Wi-N and Wi-A. (E) Senescence-associated β-galactosidase assay in cells treated with different concentration of Wi-A and Wi-N.

Three-dimensional structure of the substrate-binding domain (residues 439–597) of mortalin (PDB-id: 3N8E) was docked with Wi-A and Wi-N. As shown in Figures 3A and 3B, the amino acid residue Arg 513 of mortalin interacted with both Wi-A and Wi-N. The residue Arg 513 constitutes the carboxy-terminus region of the protein and functions as a “latch” between the “lid and cleft regions” (the substrate binding domain) that is important for chaperone function of the protein [27]. Based on these interactions, it was anticipated that the interaction of Wi-A and Wi-N with mortalin might cause an antagonistic effect on the chaperone activity of the protein. Furthermore, as summarized in Table 2, Wi-A was found to bind more efficiently with mortalin, both in terms of binding energy (exhibited a binding energy of −9.8 Kcal/mol) and affinity (Wi-A formed six hydrogen bond interactions with four amino acid residues), when compared to Wi-N that exhibited a binding energy of −8.9 Kcal/mol and formed only three hydrogen bond interactions (with three amino acid residues) (Table 2). In order to determine whether the binding of Wi-A or Wi-N to mortalin affect its stability in cells, we examined the level of mortalin in control, Wi-A and Wi-N treated cells by Western blotting using anti-mortalin antibody. As shown in Figure 3C, there was no major difference in the protein amount in control and treated cells suggesting that neither Wi-A nor Wi-N affected the expression level either by the transcription or degradation of the protein.

**Table 2:** The hydrogen bond interactions between the mortalin (PDB-id: 3N8E) and small molecules - withaferin A and withanone.

Protein	Small molecule	Hydrogen bond interactions	Distance (Å)	Binding energy (Kcal/mol)
Mortalin (PDB-id: 3N8E)	Withaferin A	Glu (483) O2-H…O	3.20	−9.8
		Arg (513) NH1-H…O4	2.97	
		Arg (513) NH1-H…O6	3.13	
		Arg (513) NH2-H…O6	2.88	
		Gly (587) N-H…O6	2.84	
		Asn (583) O6-H…O	2.61	
	Withanone	Leu(450) O4-H…N	2.64	−8.9
		Arg(513) NH2-H…O1	2.66	
		Asn(583) O-H…O1	2.96	

By an independent study, it was identified that withanone forms hydrogen bond interactions with amino acid residues Phe 272, Asp 136, Asn 139, Asp 277, Glu 270 and Arg 284 from mortalin. This resulted in the abrogation of mortalin-p53 complexes and in the activation of p53 function [34]. In view of this finding, we treated human cancer cells with various doses of Wi-N and Wi-A and examined the subcellular distribution of mortalin by immunostaining using a specific antibody. As shown in Figure 3D, 0.1 µg/ml of Wi-A caused shift in mortalin staining pattern from perinuclear (typical of cancer cells) to pancytoplasmic one (typical of normal cells) [35]–[37]. Whereas cells when treated with 0.1 µg/ml of Wi-A showed growth arrest, further increase in the treatment dose (0.5 µg/ml and higher) led to apoptosis. Interestingly, 0.1 µg/ml of Wi-N caused only a mild arrest in cancer cell growth with no change in the staining pattern of mortalin; doses such as 1.0 µg/ml or 5.0 µg/ml were required to achieve the growth arrest equivalent to phenotype observed with Wi-A (0.1 µg/ml). Consistent with this, mortalin staining pattern did not show any change at 0.1 µg/ml of Wi-N; about 30–40% of cells treated with Wi-N (5 µg/ml) showed shift in staining pattern from the perinuclear to pancytoplasmic type. These data were consistent with the predicted stronger binding of Wi-A to mortalin than of Wi-N. Furthermore, whereas 0.1 µg/ml of Wi-A was toxic to the normal cells (Figure 2) and Wi-N was tolerated even at doses 1.0 and 5.0 µg/ml. The result was attributed to the lack of mortalin-p53 interaction in normal cells [31], [37], [38]. Although specific functions of the perinuclear and pancytoplasmic mortalin have not been elucidated, a shift in mortalin staining pattern from perinuclear to pancytoplasmic type has earlier been used as a marker of induced senescence in human cancer cells [37], [39]. These data suggested that Wi-A is an efficient senescence (at low doses) and apoptosis (at high doses)-inducing reagent for cancer cells. It also caused cytotoxicity to normal cells at equivalent doses. On the other hand, weak binding of Wi-N to mortalin, as predicted by molecular docking analysis and validated by experiments, is sufficient to induce senescence in cancer cells and spare normal cells suggesting that Wi-N could be a safer drug for cancer therapy. The results were also supported by senescence-associated β-gal staining of control, Wi-A and Wi-N cells. As shown in Figure 3E, Wi-A (0.1 µg/ml) treated U2OS cells showed 60% β-gal positive cells as compared to Wi-N (0.1 µg/ml) treatment that showed staining in only 5–10% cells. Whereas cells treated with Wi-A (1 µg/ml) showed more than 90% β-gal positive cells, Wi-N doses from 5–10 µg/ml showed 50–70% β-gal positive cells, respectively. These data supported that Wi-A caused stronger cytotoxicity to cancer cells.

### Docking of Withaferin A and Withanone into p53 Structure and Experimental Examination of their Differential Effect in Human Cancer Cells

**Figure 4:**
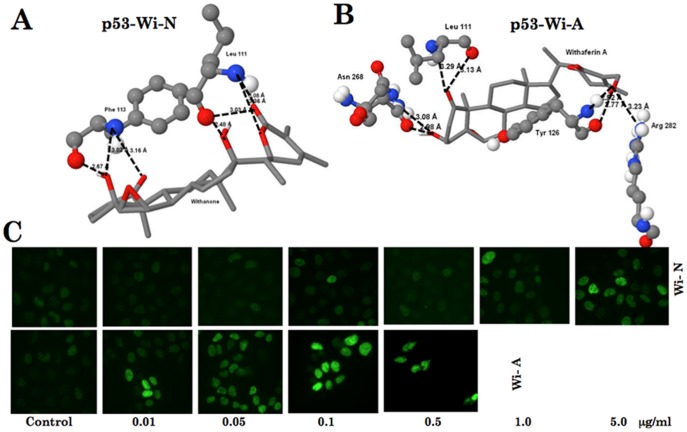
p53, a target of withanone and withaferin A. Docking of withanone (Wi-N) (A) and withaferin A (Wi-A) (B) to p53. (C) Imaging of p53 in human cancer (U2OS) cells treated with various concentrations of Wi-N and Wi-A.

p53 is a tumor suppressor protein of 53-kDa that regulates cell cycle and prevents cancer by inducing apoptosis, activating DNA repair mechanism and enhancing the activity of p21^WAF1^. It is inactivated by various mechanisms including gene mutations, deletions and binding partners in majority of cancers. p53 based therapy has been recognized as one of the core cancer therapeutic strategies in which rescuing wild type p53 activity has been predicted as an efficient way of tumor killing [40]–[43]. Since p53 was identified as one of the target genes for selective toxicity of i-Extract in human cancer cells, we performed docking analysis of Wi-A and Wi-N with p53. As shown in Figure 4A, the residue Arg 282 showed the hydrogen bond interaction with Wi-A. Interestingly, Arg 282 has been shown to be essential for the stability of protein [40]–[43] and is one of the mutation “hot spots”. When the residue Arg 282 mutates to Trp, it results in the destabilization of the H2 helix and disrupts DNA binding, resulting in a mutant type p53. The differential interaction of Wi-A and Wi-N with Arg 282 may support the previous studies. Furthermore, some studies have shown that the effects caused by V143A mutation could be corrected by Leu 111. The residues Leu 111 and Phe 113 interact with Asp 268 to stabilize the β-sandwich sheets, this in turn provides structural stability to the protein. Both Wi-A and Wi-N were seen to form hydrogen bond interactions with Leu 111 and that might support their anti-tumor activity. Although Arg 282 is not involved in the direct binding of p53 to mortalin, which involves residues 312–352 of p53 [32], it is possible that the interactions between Wi-A and Wi-N with the residue Arg 282 and Leu 111 may hinder accessibility of the binding sites of the two proteins and hence affect their binding. Analysis of the docking results (Table 3) showed that Wi-A binds more efficiently (in terms of binding affinity and energy) with p53 than Wi-N. The binding of p53 to mortalin was shown to be inhibitory for p53 functions. Release of p53 from mortalin complexes led to its translocation to nucleus and activation of p53. We examined the effect of Wi-A and Wi-N on nuclear localization of p53 in human cancer cells. As shown in Figure 4C, treatment of cells with Wi-A (0.01 to 0.05 µg/ml) caused increase in nuclear p53, associated with the induction of growth arrest. Although equivalent doses of Wi-N were ineffective, nuclear translocation of p53 occurred at 1.0 to 5.0 µg/ml treatment, and were associated with growth arrest. We examined the changes in the expression level of p21, an immediate downstream effector of p53 protein that has been established to mediate the cell growth arrest by p53, as described below.

**Table 3:** The hydrogen bond interactions between the p53 (PDB-id: 3D09) and small molecules - withaferin A and withanone.

Protein	Small molecule	Hydrogen bond interactions	Distance (Å)	Binding energy (Kcal/mol)
p53 (PDB-id: 3D09)	Withaferin A	Arg (282) NH1-H…O6	3.23	−10.18
		Tyr (126) O6-H…O	2.77	
		Tyr (126) N-H…O6	2.92	
		Leu (111) O-H…O1	3.13	
		Leu (111) N-H…O1	3.29	
		Asn (268) ND2 _(A)_-H…O2	3.08	
		Asn (268) O2-H…OD1_(A)_	2.98	
	Withanone	Leu (111) O4-H…O	2.48	−7.98
		Leu (111) O-H…O5	3.03	
		Leu (111) N-H…O5	3.08	
		Leu (111) N-H…O6	3.36	
		Phe (113) O2-H…O	2.67	
		Phe (113) O2-H…N	3.02	
		Phe (113) N-H…O1	3.16	

The symbol (A) denotes the A-conformation of the molecule.

### Docking of Withaferin A and Withanone into p21 Structure and Experimental Examination of their Differential Effect in Human Cancer Cells

**Figure 5:**
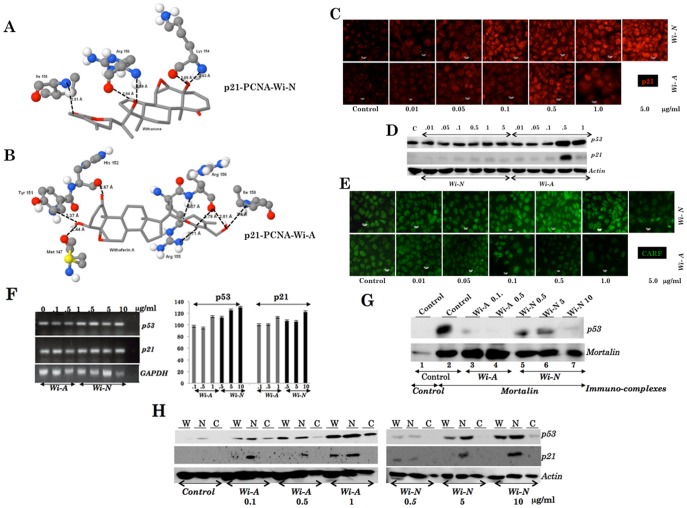
p53 downstream effector, p21, a target of withanone and withaferin A. Interaction of withanone (Wi-N) (A) and withaferin A (Wi-A) (B) with p21^WAF1^-PCNA (PDB-id: 1AXC) complex, and imaging of p21 in human cancer (U2OS) cells treated with various concentrations of Wi-N and Wi-A (C). (D) Western blot analysis of p53 and p21. (E) Immunostaining of CARF in U2OS cells treated with serial doses of Wi-N and Wi-A. (F) RT-PCR analysis of p53 and p21 expression in U2OS cells treated with Wi-A and Wi-N. (G) Co-immunoprecipitation of mortalin and p53 in control and treated U2OS cells showing decrease in p53 in mortalin immunocomplexes from the treated cells. (H) Detection of p53 and p21 in nuclear fractions of U2OS cells treated with Wi-A and Wi-N.

p21 is a p53-dependent inhibitor of cyclin-dependent protein kinases (CDK-2 and CDK-4) and controls the initiation of S phase in cell cycle [44]–[46]. In addition, it can also bind and inhibit the activity of DNA polymerase processivity factor PCNA [proliferating cell nuclear antigen] [47], [48]. Docking studies performed on the three-dimensional structure of the carboxy-terminus region of p21 (22 residues) bound to PCNA (PDB-id: 1AXC, chain B) with both the small molecules (Wi-A and Wi-N) showed that they effectively interact with the receptor molecule (Table 4). The five hydrogen bond interactions between Wi-A and the residues Arg 155, Arg 156 and Ile 158 (Figure 5A), and the three hydrogen bond interactions between Wi-N and residues Arg 156 and Ile 158 (Figure 5A) were thought to be significant, because these amino acids play a crucial role in the formation of the p21-PCNA complex [49]. Studies have shown that the residues Arg 155 and Arg 156 of p21 make hydrogen bond interactions with Glu 124 and Glu 125 of PCNA in p21-PCNA complex. Binding of Wi-A and Wi-N to one or both of these residues could influence the activity of p21. Furthermore, analysis of the docking results showed that Wi-A binds more efficiently (by forming eight hydrogen bond interactions) with p21 than Wi-N (forms five hydrogen bond interactions).

**Table 4:** The hydrogen bond interactions between the p21^WAF1^ (PDB-id: 1AXC) and small molecules - withaferin A and withanone.

Protein	Small molecule	Hydrogen bond interactions	Distance (Å)	Binding energy (Kcal/mol)
p21^WAF1^ (PDB-id: 1AXC)	Withaferin A	Met (147) O2-H…O	2.44	−7.84
		Tyr (151) N-H…O2	3.37	
		His (152) O-H…O1	2.67	
		Ile (158) N-H…O6	3.40	
		Arg (156) O6-H…O	2.81	
		Arg (156) O-H…O4	2.79	
		Arg (156) N-H…O5	3.07	
		Arg (155) NH1-H…O4	2.71	
	Withanone	Lys (154) N-H…O2	2.63	−7.84
		Lys (154) O2-H…O	2.65	
		Arg (156) N-H…O4	2.59	
		Arg (156) O4-H…O	2.84	
		Ile (158) N-H…O5	2.81	

Based on these data, we anticipated that Wi-A and Wi-N might disrupt the formation of p21-PCNA complex. We next examined p21 in human cancer cells after the treatment with either Wi-A or Wi-N. As shown in Figures 5C-5E, we found that p21 gets upregulated and translocated to nucleus in cells treated with Wi-A (0.1 to 0.5 µg/ml doses). Wi-N treated cells that showed growth arrest at doses 1.0–5.0 µg/ml. These cells showed nuclear p21 by immunostaining and increased level of expression by Western blotting suggesting that the growth arrest caused by Wi-N was mediated by an increase in p21 activity. Interestingly, cells that underwent apoptosis in response to the treatment with high dose of Wi-A showed decrease in p21 (Figure 5C and 5D) suggesting that Wi-A induced apoptosis is mediated by p21-independent additional mechanism(s). Induction of apoptosis by high dose of Wi-A treatment was also confirmed by decrease in expression of CARF (Figure 5E) that has earlier been shown to induce caspase-mediated apoptosis signaling [50] Furthermore, In order to investigate the regulation of p53 and p21 by Wi-A and Wi-N, we examined their expression in control and treated cells by RT-PCR and found that although at different doses of Wi-N (0.5, 5 and 10 µg/ml) and Wi-A (0.1, 0.5 and 1 µg/ml), both p53 and p21 were upregulated at the transcriptional level in treated as compared to the control cells (Figure 5F). We also examined the mortalin-p53 complex formation (Figure 5G) and nuclear translocation of p53 and p21 in control and treated cells (Figure 5H). As shown, p53 immunocomplexes from untreated control cells showed the presence of p53 in mortalin-immunocomplexes (Figure 5G, lane 2). Wi-A (0.1 and 0.5 µg/ml) and Wi-N (0.5, 5 and 10 µg/ml) showed decrease in p53 in mortalin complexes (Figure 5G, lanes 3–7). Consistent with these, we found that there was increase in p53 and p21 in the nuclear fractions of the treated cells (Figure 5H). Of note, 5–10 fold higher dose of withanone was required to obtain the same amount of nuclear concentration of the proteins obtained by treatment with withaferin A.

### Docking of Withaferin A and Withanone into Nrf2 Structure and Experimental Examination of their Effect in Human Cancer Cells

**Figure 6:**
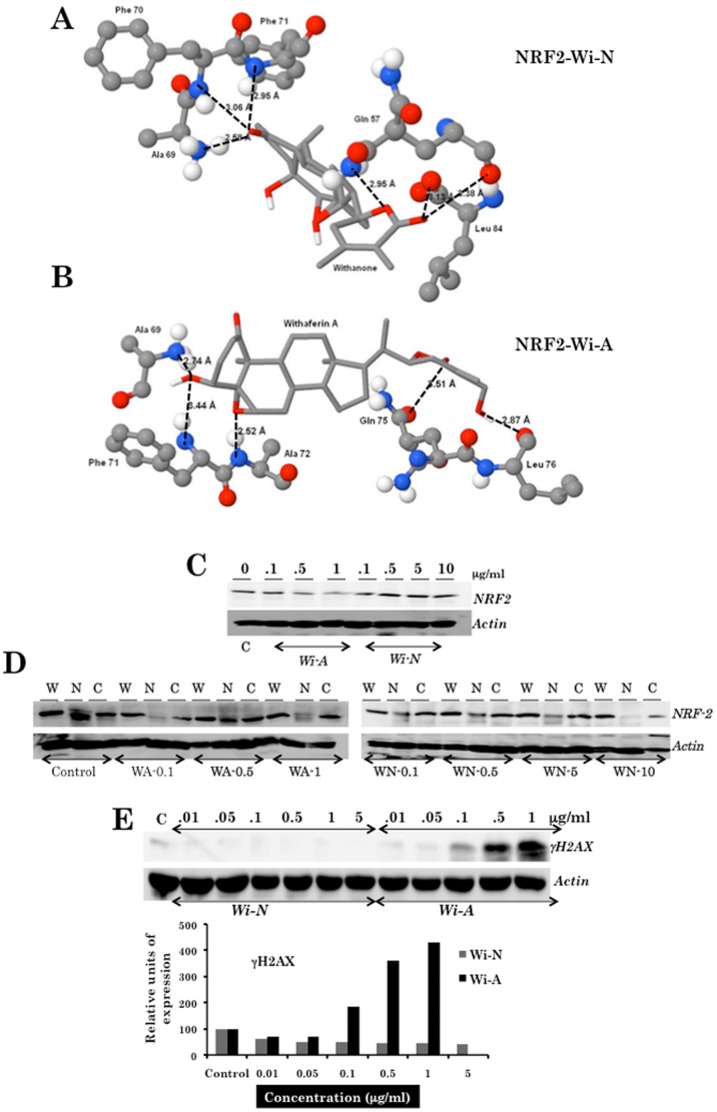
NRF2, a target of withanone and withaferin A. Docking of withanone (Wi-N) (A) and withaferin A (Wi-A) (B) with Nrf-2 (PDB-id: 2FLU), and Western blot analysis of Nrf2 in U2OS cells treated with Wi-A and Wi-N (C). (D) Detection of Nrf2 in the nuclear and cytosolic fractions of cells treated with Wi-A and Wi-N. (E) Western blot analysis of γH2AX in U2OS cells treated with Wi-N and Wi-A.

Nrf2 is a protein involved in the regulation of antioxidant response and when gets abnormal results in cancer and other inflammatory diseases [51], [52]. Keap1-Nrf2-ARE signaling plays a significant role in protecting cells from endogenous and exogenous stresses. Under normal conditions, the transcription factor Nrf2, a primary transcription factor required for the induction of a battery of phase II detoxification genes through activation of antioxidant response element (ARE), interacts with the actin-anchored protein Keap1 and is largely localized in the cytoplasm resulting in the low basal expression of Nrf2-regulated genes. However, upon recognition of chemical signals imparted by oxidative and electrophilic molecules that cause activation of MAPKinase signaling, Nrf2 is phosphorylated, released from Keap1, escapes proteasomal degradation, translocates to the nucleus, and transactivates the expression of several antioxidant and cytoprotective genes that increase resistance to oxidative stress and mitochondrial dysfunction leading to enhanced cell survival. Molecular docking studies conducted on the three-dimensional structure of Nrf2 (PDB-ID: 2FLU) showed that both Wi-A and Wi-N interact with amino acids Ala 69, Phe 71 and Gln 75 (Figures 6A and 6B) in the active site region as predicted by CASTp server [53]. Furthermore, Wi-A binds more efficiently (in terms of binding affinity) with the target protein (Table 5).

**Table 5:** The hydrogen bond interactions between the NRF-2 (PDB-id: 2FLU) and small molecules - withaferin A and withanone.

Protein	Small molecule	Hydrogen bond interactions	Distance (Å)	Binding energy (Kcal/mol)
NRF2 (PDB-id: 2FLU)	Withaferin A	Ala (69) N-H…O2	2.74	−7.87
		Phe (71) N-H…O2	3.44	
		Ala (72) N-H…O3	2.52	
		Gln (75) OE1-H…O4	3.51	
		Leu (76) O6-H…O	2.87	
	Withanone	Leu (84) O-H…O5	3.13	−6.46
		Gln (75) O-H…O5	3.38	
		Gln (75) NE2-H…O6	2.95	
		Phe (71) N-H…O1	2.95	
		Phe (70) N-H…O1	3.06	
		Ala (69) N-H…O1	2.58	

We examined Nrf2 in cancer cells treated with either Wi-A or Wi-N. As shown in Figures 6C and 6D, neither Wi-A nor Wi-N caused any significant changes in the level of expression of Nrf2. The level of NRF2 and its nuclear translocation, if any, were examined by Western blotting and immunostaining, respectively. These assays revealed neither change in the localization of NRF2 nor its induction, rather the level was decreased in the nuclear fraction (Figures 6C, 6D and data not shown). These data suggested that neither Wi-A nor Wi-N evoked Nrf2 response in cancer cells accounting for their death. Differential molecular response by Wi-A and Wi-N was further examined for another oxidative stress and DNA damage response marker protein γH2AX. As shown in Figure 6E, only the cells treated with Wi-A (0.1 mg/ml and above) showed strong induction of γH2AX protein but the cells treated with equivalent doses of Wi-N remained unaffected.

Withaferin A (Wi-A) and Withanone (Wi-N), the two steroidal lactones have closely related chemical structure but exhibit different biological activities. It has earlier been shown that Wi-A induces senescence and growth arrest at low concentration level, and apoptosis at high doses, both in normal and cancer human cells, and is an efficient inhibitor of tumor growth. Wi-N, on the other hand, showed cytotoxicity to cancer cells; the normal cells remained unaffected at the equivalent doses. In the present study, the molecular docking analyses of various gene targets, previously identified from the loss-of-function screening assays, revealed that Wi-A has high binding efficacy (in terms of energy) and interacted with many biologically significant amino acid residues of the selected gene targets. The interactions of Wi-A and Wi-N with Arg 513 of mortalin that plays an important role in chaperone activity could predict loss of such activity of the protein. Conformational changes associated with such interaction may explain shift in mortalin staining pattern and release of p53 from mortalin-p53 complexes resulting in the activation of p53 as also demonstrated in our earlier studies [33], [37]. This effect may also be mediated by binding of Wi-A and Wi-N to Arg 282 and Leu 111 of p53 resulting in change in its conformation and interaction with mortalin. Both molecular docking and experimental data have revealed that Wi-A could act as a strong inducer of oxidative stress via γH2AX pathways. Wi-N, on the other hand, demonstrated milder binding efficiency (in terms of energy) and interacted with fewer amino acid residues of the selected targets. Furthermore, whereas Wi-N induced growth arrest appeared to be mediated to a large extent by its interaction with p21, the apoptosis induced by Wi-A seemed to be independent of the p21 pathway (Figures 5C and 5D). Taking together, these findings suggested that the stronger cytotoxicity of Wi-A to cancer cells is due to its strong binding efficacy and multisite interaction. Although Wi-A acted as a powerful anti-cancer reagent, it also caused toxicity to normal cells. Wi-N, on the other hand, worked as a milder, yet effective, anti-cancer reagent and safe to normal cells. The selective activation of the molecular responses and signaling as predicted and demonstrated in this study is thought to be a basis of the differential action of these two closely related phytochemicals.

The present study revealed the critical differences in the activity of Wi-N and Wi-A that may prove as significant information for their use in cancer treatment by themselves or in combination with other modalities such as DNA damaging agents and radiation. Studies on multiple cancer cell lines, in *in vitro* and *in vivo* models, are warranted to further endorse that the care should be taken while using alternative drugs or replacing drugs on the basis of closely related chemical formulas. Furthermore, proper care of dosage of phytochemicals is crucially important for biological responses.

## Materials and Methods

### Protein Preparation

In the previous studies, mortalin, p53, Nrf-2 and p21^WAF1^ were identified as gene targets of i-Extract induced cancer cell killing [21], [22]. These proteins are known to play major role in control of cell proliferation and stress resulting in induction of cellular senescence and hence were considered as the subject of present investigation. The three-dimensional atomic coordinates of the crystal structures of the above mentioned proteins (PDB-ID: 3N8E, 3D09, 2FLU and 1AXC) were downloaded from the Protein Data Bank archive and used for docking studies. The protein structures were prepared in order to obtain the correct ionization and tautomeric states of amino acid residues. Further, the water molecules were removed and polar hydrogen atoms were added. Then, the Kollman united atom partial charges and salvation parameters were assigned. The protein preparation process resulted in a PDBQT file that contained the atomic coordinates of the protein in a format that was necessary to execute AutoGrid and AutoDock.

### Ligand Preparation

The three-dimensional structures of small molecules (crystallized and solved in our laboratory, unpublished results) were prepared by identifying the root and its expansion, as required by the docking programs. Further, the torsion angles were identified as five for Wi-A and four for Wi-N that denotes the flexibility of the ligand molecule.

### Ligand Docking

AutoDock is a tool used for predicting the interactions between the receptor (macromolecule) and the ligand molecule. AutoDock 4.2 suite was used for molecular docking analysis and the docking logs were analyzed using the graphical user interface of ADT. The Lamarckian Genetic Algorithm was used with a population of 250 dockings. Initially, the grid box was generated for the entire protein molecule, because the protein structures were not complexed with a small molecule. Further, at the end of the docking process (for each of the four proteins), a possible ligand-binding site was identified and another grid box was generated around that area. Then the final docking calculations were performed and compared with the initial docking results in order to confirm the accuracy of the predicted binding sites. The results were clustered into similar conformations based on the cluster root mean square deviation and orientation.

### Cell Culture

U2OS (osteosarcoma) and TIG (normal skin fibroblast) cells were maintained in Dulbecco’s Modified Eagle’s Medium DMEM (Invitrogen) supplemented with 10% fetal bovine serum in a humidified incubator (37°C and 5% CO_2_). Cells (40–60% confluency) were treated with different concentrations (0.01, 0.05, 0.1, 0.5, 1.0 and 5.0 µg/ml) of withaferin A (Wi-A) and withanone (Wi-N) for 48 h. These samples were then processed for different assays as follows.

### Cell Morphologic Observations and Growth Assays

The cells were grown in 12-well culture dish to about 50% confluency and then treated with different concentrations of withaferin A (Wi-A) and withanone (Wi-N). After 48–72 h, morphology of control and treated cells was recorded by phase contrast microscope (Axioplan 2 Imaging, Carl Zeiss, Inc.). Cell growth assays were performed using cell viability assay kit (MTT assay kit, Life Technologies).

### Immunostaining

Cells were cultured and treated on glass coverslips placed in 12-well culture dish. At the end of the treatment, coverslips were washed with cold phosphate-buffered saline (PBS) and the cells were fixed with pre-chilled methanol:acetone (1∶1 v/v) mixture for 5–10 min on ice. The fixed cells were washed with PBS, permeabilized with 0.2% Triton X-100 in PBS for 10 min, and blocked with 2% bovine serum albumin (BSA) in PBS for 10 min. The cells were stained with anti-p53 (DO-1), anti-p21 (C-19), anti-Nrf2 (H-300) antibodies (Santa Cruz Biotech.), anti-γH2AX (Millipore) and polyclonal anti-mortalin antibody [32]. Immunostaining was visualized by secondary staining with Alexa-488/Alexa-594 conjugated goat anti-rabbit/mouse antibody (Molecular Probes). After three to four washings with 0.2% Triton X-100 in PBS (PBST), the cells were overlaid with Fluoromount (Difco) and examined under Carl Zeiss microscope with epifluorescence optics.

### β-galactosidase (β-gal) Assay

Senescent cells were detected using the standard protocol using Senescence β-galactosidase Staining Kit (Cell Signalling). In brief, cells were washed with PBS, fixed with fixative solution for 10 min, washed again with PBS, followed by overnight incubation in staining solution supplemented with X-gal at 37°C. Stained cells were observed under the microscope.

### Separation of Nuclear and Cytoplasmic Fractions

The cytoplasmic and nuclear fractions were separated using Qproteome Nuclear Protein Kit (Qiagen). The cells, treated with different concentrations of Wi-N and Wi-A, were washed with ice-cold PBS. Cells were removed from culture plate with cell-scraper and centrifuged at 450×*g* for 5 min at 4°C. The cell pellet was gently re-suspended in 500 µL of lysis buffer NL (supplemented with Protease inhibitor solution and 0.1 M DTT) followed by 15 min incubation on ice. Thereafter, the re-suspended cells were transferred to a clean pre-chilled microcentrifuge tube. 25 µL of detergent solution NP was added to the cell suspension and vortex for 10 s at maximum speed. The cell suspension was centrifuged at 10,000×*g* for 5 min. The supernatant (cytosolic fraction) was transferred to a pre-cooled tube and stored at −80°C. The pellet (containing cell nuclei) was re-suspended in 500 µL Nuclear Protein Lysis buffer NL (supplemented with Protease Inhibitor Solution and 0.1 M DTT) by vortexing for 5 s at maximum speed. The nuclei suspension was centrifuged for 5 min at 10,000×*g* at 4°C. The supernatant was discarded and nuclear pellet was stored at −80°C for further use. The nuclear pellet was dissolved in 1X RIPA buffer (Thermo Scientific) (supplemented with Protease Inhibitor Cocktail, Roche Diagnostics) and protein estimation was done using BCA kit (Pierce Biotechnology, Inc.).

### Immunoprecipitation

Pre-clearing is performed by mixing 300 µl of NP-40 lysis buffer, specified antibody and 10 µl of Protein-A/G plus Agarose (Santa Cruz Biotechnology) on a rotor at 4° overnight. The agarose beads were washed with NP-40 lysis buffer thrice at 13,000 rpm for 2 min. Cell lysates (300 µg of protein) is added to the above and the volume is made up to 300 µl with NP-40 lysis buffer. It is rotated on a rotor overnight at 4°C. Immunocomplexes (IC) were resolved on 10% SDS-PAGE, electroblotted onto a PVDF membrane (Millipore Corporation, Billerica, MA) using a semi-dry transfer system (Biorad, Hercules, CA) and the proteins in IC were detected by Western blotting with the indicated monoclonal antibody and viewed using ECL chemiluminescence (Amersham Pharmacia Biotech, Piscataway, NJ).

### Western Blotting

The protein (20 µg, estimated by Bradford method) was separated on SDS-polyacrylamide gel and electroblotted onto a nitrocellulose membrane (Millipore) using a semidry transfer blotter. Immunoassays were done using anti-p53, anti-p21, anti-Nrf2, anti-mortalin and anti-actin antibodies. The immunocomplexes formed were visualized with horseradish peroxidase-conjugated anti-rabbit immunoglobulin G (IgG; ECL; Amersham Pharmacia Biotech, Piscataway, NJ).
